# Phytochemical Investigation and Biological Activities of *Lantana rhodesiensis*

**DOI:** 10.3390/molecules26040846

**Published:** 2021-02-05

**Authors:** Fatimata Nea, Michel Boni Bitchi, Manon Genva, Allison Ledoux, Alembert Tiabou Tchinda, Christian Damblon, Michel Frederich, Zanahi Félix Tonzibo, Marie-Laure Fauconnier

**Affiliations:** 1 Laboratory of Constitution and Reaction of Matter, UFR-SSMT, University Félix Houphouët-Boigny, 01 BP 582 Abidjan 01, Ivory Coast; bbonimichel@yahoo.fr (M.B.B.); tonzibz@yahoo.fr (Z.F.T.); 2 Laboratory of Chemistry of Natural Molecules, Gembloux Agro-Bio Tech, University of Liège, Passage des Déportés 2, 5030 Gembloux, Belgium; m.genva@uliege.be (M.G.); marie-laure.fauconnier@uliege.be (M.-L.F.); 3 Laboratory of Pharmacognosy, Center for Interdisciplinary Research on Medicines (CIRM), University of Liège, Avenue Hippocrate 15, 4000 Liège, Belgium; allison.ledoux@uliege.be (A.L.); m.frederich@uliege.be (M.F.); 4 Laboratory of Phytochemistry, Centre for Research on Medicinal Plants and Traditional Medicine, Institute of Medical Research and Medicinal Plants Studies, P.O. Box 13033 Yaoundé, Cameroon; talembert@gmail.com; 5 MolSys Research Unit, Faculty of Sciences, University of Liège, 4000 Liège, Belgium; c.damblon@uliege.be

**Keywords:** *Lantana rhodesiensis*, polyphenol content, flavonoid content, antioxidant activity, anti-malarial activity, flavones

## Abstract

*Lantana rhodesiensis* Moldenke is a plant widely used to treat diseases, such as rheumatism, diabetes, and malaria in traditional medicine. To better understand the traditional uses of this plant, a phytochemical study was undertaken, revealing a higher proportion of polyphenols, including flavonoids in *L. rhodesiensis* leaf extract and moderate proportion in stem and root extracts. The antioxidant activity of the extracts was also determined using three different assays: the radical 2,2-diphenyl-1-picrylhydrazyl (DPPH) scavenging activity, the FRAP method (Ferric-reducing antioxidant power) and the β-carotene bleaching test. The anti-malarial activity of each extract was also evaluated using asexual erythrocyte stages of *Plasmodium falciparum*, chloroquine-sensitive strain 3D7. The results showed that the leaf extract exhibited higher antioxidant and anti-malarial activities in comparison with the stem and root extracts, probably due to the presence of higher quantities of polyphenols including flavonoids in the leaves. A positive linear correlation was established between the phenolic compound content (total polyphenols including flavonoids and tannins; and total flavonoids) and the antioxidant activity of all extracts. Furthermore, four flavones were isolated from leaf dichloromethane and ethyl acetate fractions: a new flavone named rhodescine (5,6,3′,5′-tetrahydroxy-7,4′-dimethoxyflavone) (**1**), 5-hydroxy-6,7,3′,4′,5′-pentamethoxyflavone (**2**), 5-hydroxy-6,7,3′,4′-tetramethoxyflavone (**3**), and 5,6,3′-trihydroxy-7,4′-dimethoxyflavone (**4**). Their structures were elucidated by ^1^H, ^13^CNMR, COSY, HSQC, HMBC, and MS-EI spectral methods. Aside from compound **2**, all other molecules were described for the first time in this plant species.

## 1. Introduction

*Lantana rhodesiensis (L. rhodesiensis)* is an aromatic plant used in traditional medicine to treat many diseases, such as rheumatism, diabetes mellitus [1], malaria [2], cancer [3], congestive heart failure, and cardiac arrhythmia [4,5]. It is a woody herb or small shrub less than 2 m high, often with several stems, and without thorns, native to subtropical and tropical regions. *L. rhodesiensis* can be found in many African countries, such as Tanzania, Kenya, Rwanda, Ethiopia, Malawi, Cameroon, Sudan, Burkina Faso, and Côte d’Ivoire [1,6].

Several studies have already tried to correlate the traditional uses of this plant with its biological activities and chemical composition. As an example, aqueous extracts of *L. rhodesiensis* were screened for their hypoglycemic activities in alloxan-induced diabetic rats, with results confirming the antidiabetic activity of *L. rhodesiensis* when therapeutic doses were administered intra-peritoneally and orally [1]. In order to justify its traditional use for the treatment of cancer, the antiproliferative activity of *L. rhodesiensis* was evaluated. The results showed that *L. rhodesiensis* is not genotoxic and that this plant induces a strong antiproliferative effect against cancer cells in vitro. The high antioxidant activity of *L. rhodesiensis* methanol extracts [3] and decoctions [7] was also highlighted using DPPH method. In those studies, the methanol extracts contained high quantities of tannins and flavonoids and the decoctions were characterized by high total phenolic contents with low flavonoid quantities. *L. rhodesiensis* also showed significant repellency against *Anopheles gambiae sensu lato* Giles, the main vector of malaria in Africa [8]. Leaf essential oils from *L. rhodesiensis* have been extensively studied and shown to possess robust anti-inflammatory and antioxidant activity [9], which originates from their high content of phenolic compounds [10]. Studies have also shown that *L. rhodesiensis* contains triterpenes, steroids, phenols, alkaloids, polyphenols including flavonoids, and tannins [1,2,3,4,11]. Two polymethoxyflavones, 5,6,7,3′,4′,5′-hexamethoxyflavone and its analogue 5-hydroxy-6,7,3′,4′,5′-pentamethoxyflavone, were isolated from the whole plant of *L. rhodesiensis* [12].

The main purpose of the present research was to correlate the traditional medicine uses of *L. rhodesiensis* for treating rheumatism and malaria with the phytochemical composition of *L. rhodesiensis* extracts obtained from each plant organ and with their antioxidant and anti-malarial activities. The different plant organs were considered separately in order to determine the most active part of the plant. Finally, four major flavonoids were isolated from *L. rhodesiensis* leaves and their structures were determined, as well as their antioxidant activities.

## 2. Results and Discussion

### 2.1. Phytochemical Screening

#### 2.1.1. Determination of Phytochemical Classes

The results of the qualitative phytochemical study of *L. rhodesiensis* organs (Table 1) showed that the leaf, stem, and root extracts of *L. rhodesiensis* contained polyphenols including flavonoids and tannins. Terpenes, sterols, saponins, and alkaloids were also detected in all the organs, while leuco-anthocyanins and anthocyanins were too low to be detectable. The results also highlighted considerable differences in the phytochemical classes found in the different plant organs, as the assays indicated higher levels of flavonoids and polyphenols in leaves than in stems and roots. Moreover, the results also indicated higher proportions of saponins in roots than in leaves and stems. The phytochemical classes detected in the leaf extract are in agreement with those already described in an aqueous leaf extract [1]. Moreover, the realized assays indicated a higher proportion of tannins in the methanolic extracts from the aerial parts (stems and leaves) in comparison with sterols/triterpenes, flavonoids, and saponins [3]. This is the first systematic phytochemical screening of *L. rhodesiensis* stems and roots. 

#### 2.1.2. Polyphenolic Compound Quantification

Quercetin (coefficient of determination (R²) = 0.9996) and gallic acid (R² = 0.9975) calibration curves were performed in order to determine the phenolic compound concentrations in the extracts. Total polyphenol contents (Table 2) ranged from 153.37 ± 0.61 to 273.27 ± 0.48 mg gallic acid equivalents (GAE)/g extract; the highest content was obtained with the leaf extract. For the total flavonoid assay, the contents ranged from 34.87 ± 0.34 to 110.54 ± 0.46 mg quercetin equivalents (QE)/g extract. Similarly, the leaf extract had the highest content, showing that *L. rhodesiensis* leaves are richer in polyphenols including flavonoids and tannins than the stems and roots. The lowest contents of total polyphenols and total flavonoids were observed in the root extract. 

The determination of phenolic compounds in an aqueous extract of *L. rhodesiensis* leaves had been performed previously. The results obtained in that study showed that the amount of phenols (685.25 ± 30.77 mg GAE/g) was higher than that of tannins (323.61 ± 61.54 mg GAE/g) and flavonoids (187.33 ± 54.97 mg GAE/g) [1]. In addition, another study showed the total phenol (210.55 ± 7.5 mg GAE/g) and flavonoid (50.09 ± 1.9 mg QE/g) composition of the methanolic extract of leafy stems of *L. rhodesiensis* [11]. These results cannot be directly compared to those of the present study as the extracts, organs used, and the methods applied for the different tests are not the same. However, taking into account data from the literature, it can be said that the aqueous extract of *L. rhodesiensis* leaves is richer in phenolic compounds than the hydro-methanolic extract. On the other hand, the amount of total phenolic compounds in the methanolic extract of leaves and stems is lower than that of the leaf extract in our study. 

A study using the same method to determine the total polyphenol content was carried out on leaf methanolic extracts of different *Lantana camara* varieties. Although it is not the same species, the results of two varieties (225.15 ± 12.52 and 232.99 ± 15.97 mg GAE/g extract) were found to be similar to those of the present study [13], highlighting the considerable interest in leaves from plants of the genus *Lantana* when searching for a source of polyphenolic compounds. 

The protective effect of polyphenols has been attributed to their antioxidant properties, which can prevent molecular oxidative damage and cellular disorders leading to various pathologies such as cancer, Alzheimer’s disease, type 2 diabetes, and cardiovascular and neurodegenerative diseases. Polyphenols are also capable of reducing other risk factors for cardiovascular disease involved in metabolic syndrome (hyperglycemia, high lipid levels, insulin resistance, abdominal obesity, and high blood pressure) [14].

### 2.2. Antioxidant Activity

The antioxidant activity of the leaf, stem, and root of *L. rhodesiensis* extracts was evaluated using three different methods on methanolic extracts at different concentrations (200–1000 µg/mL). Ascorbic acid was used as a standard and its activity was evaluated under the same conditions as the extracts. 

**Figure 1:**
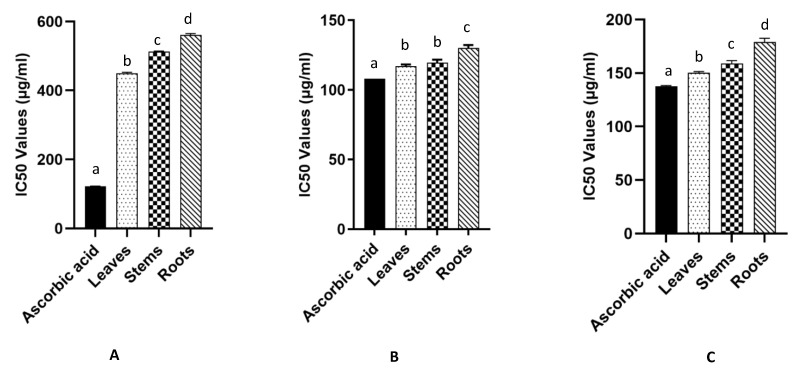
Antioxidant activity values of the methanolic extracts of *L. rhodesiensis* organs and ascorbic acid, (**A**). DPPH method, (**B**). FRAP (Ferric-reducing antioxidant power) method and (**C**). β-carotene method. Each result is the average of three values (*n* = 3). Histograms that do not share any letters are significantly different (*p*-value < 0.05).

The results of the DPPH radical scavenging test (Figure 1) show that the root extract had the lowest antioxidant activity (50% inhibition concentration, IC_50_ value: 561.36 ± 3.93 µg/mL), with higher antioxidant properties in the leaf (449.53 ± 0.56 µg/mL) and stem (512.81 ± 1.41 µg/mL) extracts. The ascorbic acid standard had an IC_50_ value of 122.09 ± 0.56 µg/mL. 

The ability of phenolic compounds to reduce Fe^3+^ ions to Fe^2+^ was measured using the FRAP (Ferric-reducing antioxidant power) method. The results show that the leaf and stem extracts had low IC_50_ values (117.08 ± 1.1 µg/mL and 119.57 ± 2.17 µg/mL, respectively), similar to that of the ascorbic acid standard (108.01 ± 0.01 µg/mL), which confirms the ability of the extracts to reduce Fe^3+^ ions, to a greater extent than the root extract (130.04 ± 2.19 µg/mL). The same trend was highlighted with the β-carotene, test as the leaf and stem extracts had low IC_50_ values (150.18 ± 1.21 µg/mL and 158.91 ± 2.65 µg/mL, respectively), similar to that of ascorbic acid (IC_50_ = 137.55 ± 0.75 µg/mL), while the root extract IC_50_ was higher (178.92 ± 3.56 µg/mL). 

Results obtained here are supported by a precedent study, showing that *L. rhodesiensis* methanolic extract dissolved in DMSO displayed a strong DPPH antioxidant activity, IC_50_ value of 5.96 ± 0.40 mg/mL [3]. The IC_50_ values obtained in the present study are different to that previous assay, but the methods used in those two studies were sensibly different (different plant parts, extracts preparation methods, dilution solvent, concentrations, volumes used, incubation time, etc.). In addition, a study has shown that using the same DPPH method, EtOH extract from the leaves of *Lantana montevidensis* showed lower antioxidant activity (IC_50_ = 290.5 ± 1.97 µg/mL) than aqueous extract (IC_50_ = 108.2 ± 3.4 µg/mL) [15]. Methanol extracts of leaves and flowers from *Lantana camara* were also already tested for their antioxidant potential, both extracts exhibiting high antioxidant and free radical scavenging activities with relatively stronger antioxidant activity in the case of whole flower extracts [16].

These three antioxidant tests show that the leaf and stem extracts of *L. rhodesiensis* have robust antioxidant activity, with interesting perspectives for their potential valorization as pharmaceuticals. 

The therapeutic effects of medicinal plants are generally attributed to their phytochemicals. Specifically, many studies have correlated the antioxidant activity of plant extracts with the presence of phenolic compounds [17,18,19,20], as they are one of the main groups of molecules that act as primary antioxidants or free radical terminators [21]. The antioxidant potential of phenols is conferred by their hydroxyl (OH^−^) group [18], which is directly linked to an aromatic hydrocarbon ring. This allows them to easily donate electrons to free radicals, and thus regulate their threat to living cells [22]. Generally, antioxidants (vitamins C, E, carotenoids, polyphenols) are important for good bone health. They neutralize reactive particles called free radicals that are associated with all inflammatory and painful phenomena [23]. The results obtained here show the high antioxidant properties of *L. rhodesiensis* extracts. More specifically, important quantities of phenols were highlighted in the leaf extract, showing higher antioxidant properties compared to the stem and root extracts. The differences between the values are in order with their phenolic content. 

The results of the DPPH test showed a considerable difference between the antioxidant activity (IC_50_) of the standard and each different organ extract studied, greater than the results of the other tests (FRAP and bleaching of β-carotene), where minor differences were highlighted. This may have been influenced by the method or test used for the evaluation of antioxidant activity, because each test has its specificities. The DPPH method is based on the measurement of antioxidant scavenging capacity towards the stable radical 2,2-diphenyl-1-picrylhydrazyl (DPPH). This method offers an easy and quick way to evaluate the anti-radical activities of antioxidants, since the radical compound is stable and does not have to be generated as in other radical scavenging tests [24]. The FRAP method is based on the ability of an antioxidant to transfer an electron to reduce any compound, including metals, carbonyl groups and radicals [25]. As for the β-carotene bleaching method, it is based on the ability of an antioxidant to neutralize free radicals generated by linoleic acid and to prevent the oxidation of β-carotene [26]. Indeed, phenolic compounds exert their antioxidant activity by several mechanisms, including the donation of hydrogen atoms to free radicals, or the trapping of other reactive species such as OH^−^, NO^2−^, N_2_O_3_, ONOOH, and HOCl. Some phenolic compounds, mainly di- and polyphenols, can react with O^2−^ or bind to transition metal ions (especially iron and copper). This often results in weakly active forms to promote free radical reactions [27,28].

**Figure 2:**
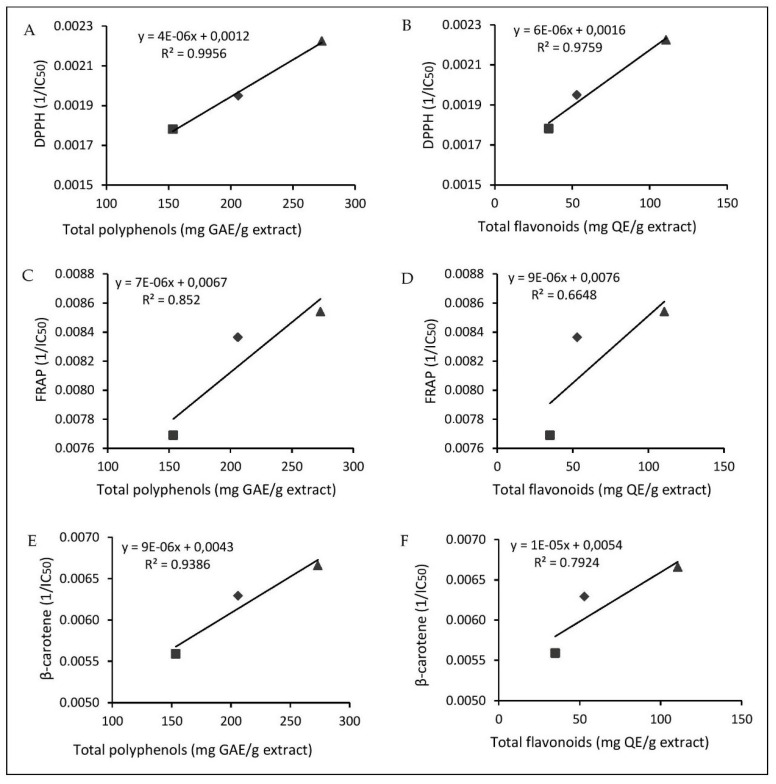
(**A**). Correlation between the total polyphenolic content and DPPH activity; (**B**). correlation between the total flavonoid content and DPPH activity; (**C**). correlation between the total polyphenolic content and FRAP activity; (**D**). correlation between the total flavonoid content and FRAP activity; (**E**). correlation between the total polyphenolic content and bleaching activity of β-carotene; (**F**). correlation between the total flavonoid content and bleaching activity of β-carotene; r: correlation coefficient and R^2^: determination coefficient; roots (■), stems (⬥), leaves (▴).

Phenols play important roles in plants, such as protection against herbivores and insect pathogens. They are involved in cementing the material linking phenolic polymers to cell wall polysaccharides [29]. In addition, they play a role in the regulation of cell growth and division [13,30]. Flavonoids are the most common and most important group of naturally occurring phenolic compounds, probably because of their wide range of functions. Flavonoids generally act through a scanning or chelation process. Flavonoids act as antioxidants by breaking radical chains in more stable products in the membranes of liver microsomes. They also play an important role in instinctive protection against oxidative stress [21,31,32,33]. In the present study, a positive linear correlation was established between the content of phenolic compounds (total polyphenols including flavonoids and tannins; and total flavonoids) and the antioxidant activity of all extracts (Figure 2). The Pearson’s correlation coefficient (r) and the coefficient of determination (R^2^) were higher (r = 0.9978, R^2^ = 0.9956) between the total polyphenolic content and DPPH activity than those of the total polyphenolic content and bleaching activity of β-carotene (r = 0.9688, R^2^ = 0.9386), followed by the total polyphenolic content and FRAP activity (r = 0.9230, R^2^ = 0.852). The correlation between the total flavonoid content and antioxidant capacity (DPPH test) was even higher (r = 0.9879, R^2^ = 0.9759). A moderate correlation (r = 0.8902, R^2^ = 0.7924) was observed for the total flavonoid content and bleaching activity of β-carotene. For FRAP activity, the correlation was lower (r = 0.8153, R^2^ = 0.6648). 

The correlation between total polyphenols and antioxidant activity was the strongest, indicating that a high phenolic content correlates with higher antioxidant activity. Phenolic compounds are produced differently depending on the plant species [34]. In addition, environmental factors, such as the drying technique, storage conditions, and the plant organ used as the source and the moisture content are parameters that could influence the phytochemical content of a plant [35,36]. Furthermore, the extraction process appears to affect the total phenolic content and antioxidant activity of the plants [3,37,38,39,40,41]. 

A positive linear correlation was also established between the three different methods used to evaluate antioxidant activity in this study. The Pearson correlation coefficient (r) for the DPPH and β-carotene assays (0.9558) was higher than that of the DPPH and FRAP assays (0.9140). However, the correlation between the FRAP method and the bleaching of β-carotene had a coefficient of 0.9929. These results indicate that the antioxidant activity values tested by the three different methods are highly correlated. Those results were expected as several others studies on plant extracts have confirmed the relationship between antioxidant activities and polyphenolic compounds [42,43,44].

### 2.3. Anti-Malarial Activity

The hydro-methanolic extracts from the different *L. rhodesiensis* organs were tested on a chloroquino-sensitive strain (3D7) of *Plasmodium falciparum* in order to evaluate their in vitro anti-malarial activity. Artemisinin was used as a positive control. The concentration that inhibited 50% of the strain (IC_50_) was determined using sigmoidal curves for each extract (Table 3). The hydro-methanolic leaf extract was found to be active against *Plasmodium falciparum* strain 3D7, while the stem and root extracts were inactive. These results highlight, for the first time, the possible value of *L. rhodesiensis* leaves in traditional medicine for the treatment of malaria. *L. camara* leaves, a plant of the same genus, has been shown to have an IC_50_ value similar to that found in this study [45,46], highlighting the interest of this plant genus for the treatment of malaria and encouraging further studies.

Some studies argue that major phytochemical groups such as flavonoids, tannins, saponins, coumarins, alkaloids, triterpenes, sesquiterpenes and steroids [47,48,49] may be responsible for the anti-malarial activity observed in some plants. As an example, *L. camara* aqueous and ethanolic leaf extracts have shown antimalarial activity close to that of the standard drug chloroquine. In that study, alkaloids, cardiac glycosides, saponins, carbohydrates, flavonoids, steroids, tannins, and terpenoids were present in the different extracts [50]. These phytochemical groups with anti-malarial potential are present in the leaves of *L. rhodesiensis*, as previously described.

### 2.4. Determination of the HPLC-PDA (Photodiode Array Detector) Polyphenol Profile Leaf, Stem, and Root Extracts

**Figure 3:**
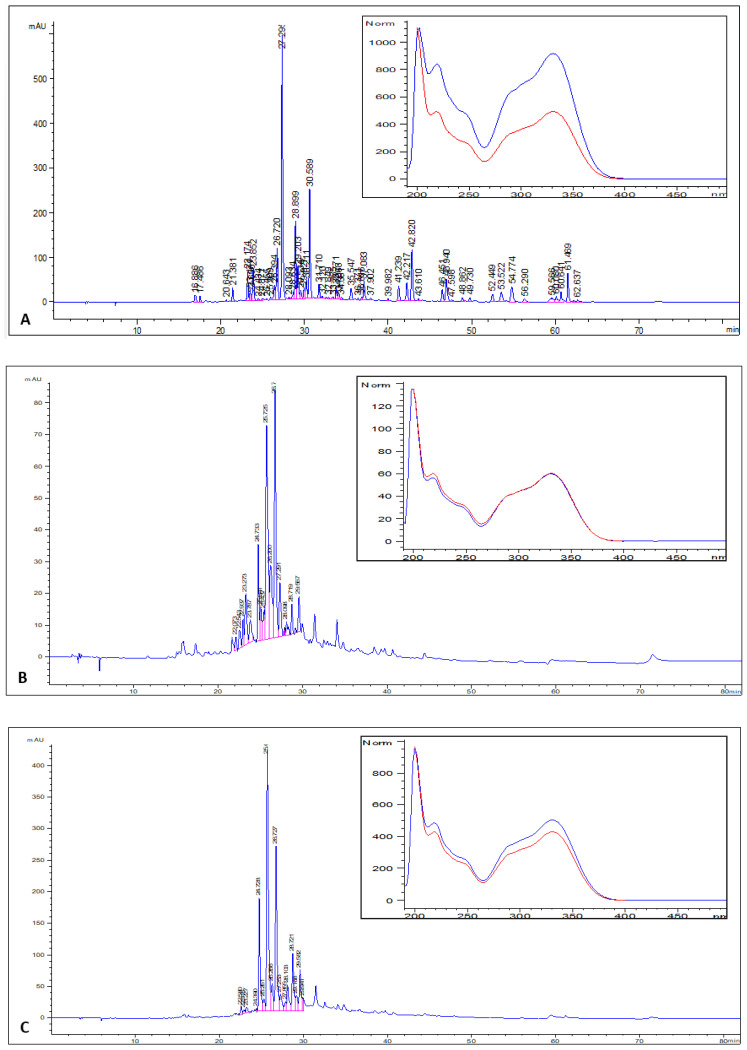
HPLC-PDA (photodiode array detector) chromatograms of the chemical profiles of polyphenols occurring in the studied extracts of leaves (**A**), stems (**B**), and roots (**C**). For each extract, the insert presents the PDA spectrum of the major peak and the PDA spectrum of the acteoside reference (in red).

The *L. rhodesiensis* leaf, stem, and root extracts were analyzed by HPLC-PDA. The major phenolic compounds were identified in each extract (Figure 3) based on their retention index and their PDA spectrum with comparison to a library. Results showed that all extracts were characterized by high quantities of isomers of acteoside, a phenolic molecule well known for its wide range of biological properties including anti-inflammatory, antioxidant and hepatoprotective activities [51,52,53,54,55].

### 2.5. Structural Elucidation

Compound **1** was obtained as yellow needles. The protonated mass, measured by LC/MS in positive mode electrospray ionization, was 346.9 [M + H]^+^, corresponding to the formula C_17_H_14_O_8_. The ^1^H- and ^13^C-NMR data for compound **1** were quite similar to those of compound **4**. In the ^1^H NMR spectrum of compound **1** (Table 1), a signal at δ_H_ 7.12 (2H, s) was attributed to two protons (H-2′, H-6′) of the B ring indicating oxygenation at C-3′, C-4′ and C-5′. Two singlets at δ_H_ 6.81 (1H) and at δ_H_ 6.59, were assigned to the H-8 and H-3 protons, respectively. These data as well as the intense signals at d 3.99 and 3.95 (both 3H, s), relative to two OCH_3_ groups, suggested presence of a tetrahydroxyflavone with two additional methoxyl group substitutions [56,57]. The ^13^C-NMR spectrum of compound **1** (Table 1) shows values between 130–155 suggesting an oxygenated A-ring. After careful analysis of 2D NMR, the hydrogen group at C-5’ in 3 was replaced by a hydroxy group in 1. So, in the HMBC spectrum, cross-peaks disclosing the bonding site of each methoxyl were observed: δ_H_ 3.99 correlated with δ_C_ 154.4 (C-7), and δ_H_ 3.95 correlated with δ_C_ 148.5 (C-4′). Correlations were also observed between H-3/C-1′, C-2, C-4 and C-10, H-8/C-6, C-7, C-9 and C-10, H-2′ and H-6′/ C-2, C-1′, C-2′, C-4′, C-5′, C-6′. Consequently, the structure of compound **1** was determined to be the new 5,6,3′,5′-tétrahydroxy-7,4′-dimethoxyflavone, named rhodescine (Figure 1).

**Figure 4:**
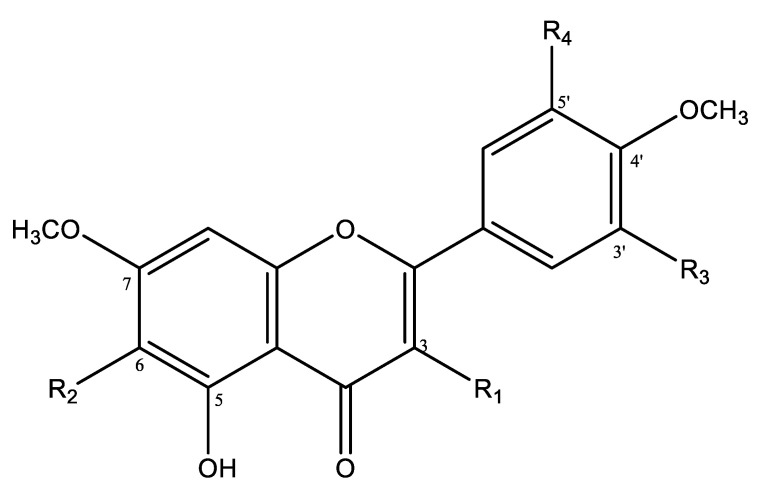
Molecular structure of purified flavones.

Compound **2**, a white amorphous powder, possessed a molecular formula of C_20_H_20_O_8_ based on the protonated ion peak at *m*/*z* 388.9 by LC/MS, indicating seven degrees of unsaturation. The ^1^H-NMR (proton nuclear magnetic resonance) (Table 4) displayed resonances for three singlets at δ_H_ 7.12, 6.64, and 6.61 ppm, suggesting aromatic ring hydrogens, and five singlets between δ_H_ 3.8 and 4.1 ppm, integrating for the three protons characteristic of a methoxy group. The ^13^C-NMR (carbon-13 nuclear magnetic resonance, J-modulated) data exhibited in total 20 carbon resonances attributed to one ketone carbonyl carbon (δ_C_ 182.8), 11 quaternary carbons (singlet, δ_C_ 165–106), four carbons (doublets, δ_C_ 105–90) suggesting C-H bonds and five carbons (singlet, δ_C_ 56–62) characteristic of carbons linked to a methoxy group. Thus, the structure of compound **1** was established as 5-hydroxy-6,7,3′,4′,5′-pentamethoxyflavone (Figure 4). This result was compared to [58].

Compound **3** was found as colorless needles. Its molecular formula was C_19_H_18_O_7_ according to the protonated ion peak at *m*/*z* 358.1 [M + H]^+^. The ^1^H-NMR spectrum exhibited signals for five aromatic ring hydrogens (δ_H_ 6.76 (1H, s), 6.89 (1H, s), 7.14 (1H, d, *J* = 8.5), 7.55 (1H, d, *J* = 2.2) and 7.68 (1H, dd, *J* = 8.5; 2.1)) and four methoxy groups [δ_H_ 3.85 (3H, s), 4.00 (3H, s), 3.93 (3H, s) and 3.96 (3H, s)] (Table 4). The ^13^C-NMR spectrum of compound **2** exhibited 19 carbon resonances (Table 4). The ^13^C-NMR data for compound **3** were quite similar to those of compound **2**. However, on the ^13^CNMR spectrum of compound **3**, there were four signals characteristic of the carbons of the methoxy group. Compound **3** was identified as 5-hydroxy-6,7,3′,4′-tétraméthoxyflavone (Figure 4). The data were compared to [59].

Compound **4** was obtained as yellow needles. The high-resolution mass spectrum of compound **3** in positive mode electrospray ionization generated a protonated ion peak at *m*/*z* 331.0862. This is compatible with the elemental composition C_17_H_14_O_7_. The ^1^H-NMR spectrum showed signals for five aromatic rings (δ_H_ 6.67 (1H, s), 6.85 (1H, s), 6.94 (1H, d, *J* = 8.3), 7.54 (1H, dd, *J* = 8.3–2.13), and 7.52 (1H, d, *J* = 2.13)) and two methoxy groups (δ_H_ 4.00 (3H, s) and 3.97 (3H, s)) (Table 4). The ^13^C-NMR spectrum of compound **3** exhibited in total 17 carbon resonances (Table 4) attributed to one ketone carbonyl carbon (δ_C_ 182.9), 10 quaternary carbons (δ_C_ 167–104), five carbons (δ_C_ 122–92) suggesting C-H bonds and two carbons of methoxy groups (δ_C_ 58.8 and 57.5). Compound **4** was characterized as 5,6,3′-trihydroxy-7,4′-dimethoxyflavone. The results were compared to [57].

All data of compounds **2**, **3,** and **4** were in good agreement with the respective literature data.

Compound **2** (5-hydroxy-6,7,3′,4′,5′-pentamethoxyflavone) has been previously reported in extracts obtained from the whole *L. rhodesiensis* plant. This molecule shows interesting anti-proliferative and pro-apoptotic properties [12]. Compounds **3** and **4** were already reported in various plant organs from other genera [57,59,60,61,62,63,64,65], but were observed here for the first time in *L. rhodesiensis* (Figure 4).

In order to explain if the high antioxidant properties of *L. rhodesiensis* leaf extracts originates from the presence of those molecules in high proportions, the antioxidant activities of purified compounds **1**, **2**, **3** and **4** were evaluated in the present study. The results showed that at a concentration of 1 mg/mL, compound **1** (97.92 ± 0.20%) inhibited DPPH better than compounds **2** (0.57 ± 0.04%), **3** (1.98 ± 0.64%) and **4** (61.77 ± 3.53%). The inhibition by compound **1** was similar to that of the standard drug used (ascorbic acid; %I = 98.50 ± 0.56).

## 3. Materials and Methods

### 3.1. Plant Materials

Leaves, stems, and roots of *Lantana rhodesiensis* (*L. rhodesiensis*) were collected from the north of Côte d’Ivoire at Kapélé (9°25′60′′ N, 5°42′0′′ W). Sample collection occurred in the morning, from 9:00 to 12:00 a.m. The plant material was identified by Professor Ake Assi and a voucher specimen (N° UCJ017435) was deposited at the Centre National de Floristique (CNF, Abidjan, Côte d’Ivoire). Each plant organ was dried during one week at room temperature (25°C) and was subsequently ground into a fine powder using mechanic ball mill, type BB-27 (E2ME). The final particle size was from a few tens to a few hundred micrometers and the moisture content was 8.5 ± 0.18% for leaves; 7.52 ± 0.64% for stems; and 6.01 ± 0.09% for roots. To determine the moisture content, the sample (organ powder) was weighed to the nearest 10 mg and dried in a drying oven at 70 °C. After 48 h, the moisture content was determined as followed:

$$\%\;Moisture=\frac{m1-m2}{m1}\times 100$$

where *m*1 is mass of the organ powder before drying and *m*2 is mass of the organ powder after drying (*n* = 3).

### 3.2. Reagent and Solvents

All reagents and solvents were either HPLC or analytical grade. Moreover, 2,2-diphenyl-1-picrylhydrazyl (DPPH), gallic acid (98%), quercetin (98%), artemisinin (98%), Folin–Ciocalteu reagent, ascorbic acid, β-carotene, and linoleic acid were purchased from Sigma-Aldrich (St. Louis, MO, USA). Anhydrous sodium sulfate, potassium ferricyanide, trichloroacetic acid (TCA), ferric chloride, sodium chloride, potassium chloride, disodium hydrogen phosphate, potassium dihydrogen phosphate, and hydrochloric acid were bought from VWR Chemicals (Radnor, PA, USA). Hexane, dichloromethane (CHCl_3_), ethyl acetate (EtOAc) and methanol (technical and HPLC) were purchased from VWR International (Fontenay-sous-Bois, Val-de-Marne, France).

### 3.3. Determination of Phytochemical Classes

The different groups of compounds (sterols/terpenes, polyphenols, flavonoids, tannins, alkaloids, saponins, leuco-anthocyanins, and anthocyanins) present in *L. rhodesiensis* leaf, stem, and root powders or extracts were identified using the methods described by Bekro et al., Bidie et al., and Nineza Claire and Nkengurutse Jacques [61,62,63].

To highlight sterols and terpenes, the reagent of Liebermann was used. Five mL of each organ extracts were evaporated on a water bath (100 °C). The residue was dissolved in 1 mL of acetic anhydride and 0.5 mL of concentrated sulfuric acid was added. The appearance of a purple and violet ring at the interphase, turning blue and then green, indicated a positive reaction. The positive standard used is the cholesterol.

In order to highlight polyphenols, the reaction with ferric chloride (FeCl_3_) was used. To 2 mL of each extract, a drop of 2% ferric chloride alcoholic solution was added. In the presence of polyphenol derivatives, ferric chloride causes the appearance of a dark blue-blackish or green coloration. The control is carried out with the alcoholic solution of gallic acid.

To highlight flavonoids, the “cyanidin” reaction was used. Two mL of each extract were evaporated and the residue was taken up in 5 mL of hydrochloric alcohol diluted twice. Then, three magnesium shavings were added and a pinkish-orange or purplish coloration was observed. By adding three drops of isoamyl alcohol, the coloration was intensified. This confirmed the presence of flavonoids. An alcoholic solution of quercetin was used as a control.

The leuco-anthocyanins were characterized by performing the same reaction as for the identification of flavonoids without the addition of magnesium shavings by heating for 15 min in a water bath. The appearance of a cherry-red or purplish coloration indicates the presence of leuco-anthocyanins.

To characterize anthocyanins, 5 mL of sulfuric acid and then 5 mL of ammonium hydroxide are added to 5 mL of the extracts. If the coloration is accentuated by acidification and then changes to purplish blue in basic medium, the presence of anthocyanins can be concluded.

Catechic tannins were identified by Stiasny reagent (formol 30%, concentrated HCl: 1/0.5). Five mL of each extract was evaporated. After 15 mL of Stiasny reagent were added to the residue. The mixture was then kept in a water bath at 80 °C for 30 min. The observation of a large flaky precipitate characterized the catechin tannins. The obtained solution was filtered and the collected filtrate was saturated with sodium acetate. The addition of three drops of 2% FeCl_3_ caused the appearance of an intense blue-black coloration, indicating the presence of gallic tannins. An alcoholic solution of gallic acid was used as a control.

In order to highlight alkaloids, the Dragendorff (iodobismuthate) reagent was used. Six mL of each extract were evaporated to dryness. The residue was taken up again in 6 mL of alcohol at 60 °C. The addition of two drops of the Dragendorff reagent to the alcohol solution caused a precipitate or an orange coloration and indicated a positive reaction.

To highlight the saponins we used the method of foam appearance by agitation. A height of persistent foam, higher than 1 cm indicates the presence of saponosides.

### 3.4. Extract Preparation for the Determination of Total Phenolic and Flavonoid Contents and Tests for Biological Activity

A methanol:water (50:50, *v*/*v*) extract was obtained by stirring 100 g of each sample with 1.5 L of the solvent mixture at 25 °C and 150 rpm for 48 h. The extract was then filtered twice through cotton and once through WATTMAN 3 mm filter paper. The solvent was then evaporated at 40°C using a rotary evaporator and the residue was subsequently lyophilized. The obtained powder was used to carry out the biological tests. The extracts were prepared on the basis of the method described by MacDonald et al. [18].

### 3.5. Determination of the Total Phenolic Content

The total phenolic content of the extracts was evaluated using the Folin-Ciocalteu method according to the Shahidi and Naczk procedure [66] with MacDonald et al. modifications [18]. Briefly, a gallic acid calibration curve was established (0, 50, 100, 150, 200, 250 mg/L) in methanol:water (50:50, *v*/*v*). Leaf, stem and root extracts were prepared in methanol:water (50:50, *v*/*v*) at a concentration of 3 mg/mL. Then, 0.5 mL of each sample or phenolic standard was mixed with 2.5 mL of Folin–Ciocalteu reagent (diluted 1:10 with distilled water) and 2 mL of aqueous sodium carbonate solution (1 M). The tubes were allowed to stand for 15 min at room temperature before the absorbance of the mixture was measured at 765 nm using a spectrophotometer. All determinations were performed in triplicate.

The total phenolic content was calculated as gallic acid equivalents (GAE) by the following:

$$T=C\times \frac{V}{M}$$

where *T* is the total phenolic content in mg/g of the extracts as GAE, *C* is the concentration of gallic acid established from the calibration curve in mg/mL, *V* is the volume of the extract solution in mL and *M* is the weight of the extract in g.

### 3.6. Determination of the Total Flavonoid Content

The total flavonoid content of the extract was determined as previously described [31,42,67]. Different concentrations (0.01–0.2 mg/mL) of quercetin, the standard molecule, were prepared in methanol. Organ extracts were also diluted in methanol (3 mg/mL). Then, 0.5 mL of methanolic samples and standards was added to 0.5 mL of aluminum chloride 10% (*w*/*v*). The same volume of sodium acetate (1 M) was added to the solution, which was then brought up to 3500 µL with distilled water. After incubation at room temperature for 30 min, the absorbance was measured at 415 nm. All determinations were carried out in triplicate. The total flavonoid content (TFC) is presented as mg of quercetin equivalents (QE) per gram of the extract.

The total flavonoid content was calculated as quercetin equivalents (QE) by the following:

$$T=C\times \frac{V}{M}$$

where *T* is the total flavonoid content in mg/g of the extracts as QE, *C* is the concentration of quercetin established from the calibration curve in mg/mL, *V* is the volume of the extract solution in mL and *M* is the weight of the extract in g.

### 3.7. Antioxidant Activity

#### 3.7.1. DPPH Radical Scavenging Assay

The antioxidant activities were measured in terms of hydrogen-donating or radical-scavenging ability, using the stable radical 2,2-diphenyl-1-picrylhydrazyl (DPPH) as a reagent [67]. To do so, various concentrations (200, 400, 600, 800, 1000 µg/mL) of each *L. rhodesiensis* organ extract and ascorbic acid were prepared in methanol. Then, 50 µL of each sample concentration was added to 2 mL of a 0.004% (*w*/*v*) DPPH methanolic solution. After 30 min of incubation at room temperature in the dark, absorbance was measured at 517 nm using an Ultrospec UV-visible dual beam spectrophotometer (GE Healthcare, Cambridge, UK). A blank sample containing the same amount of methanol and DPPH solution was used as the negative control. All determinations were performed in triplicate.

The inhibition percentage (%I) of the DPPH radical by the samples was calculated according to the formula [68]:

$$\%I=\frac{Ab-Aa}{Ab}\times 100$$

where *Ab* is the absorbance of the blank sample and *Aa* is the absorbance of the test sample.

The inhibition percentage was plotted versus the sample concentration to obtain the IC_50_ index.

#### 3.7.2. Reducing Power

The reducing power of the extracts and a standard (ascorbic acid) was determined by mixing 1 mL of the extract or standard at different concentrations in methanol (200 to 1000 µg/mL) with 1 mL of phosphate buffer (0.2 M, pH 6.6) and 1 mL of potassium ferricyanide [K_3_Fe(CN)_6_] solution (1%, *w*/*v*). The mixture was incubated at 50 °C for 20 min. After incubation, 1 mL of trichloroacetic acid (TCA) (10% *v*/*v*) was added to the solution to stop the reaction. This solution was then centrifuged at 3000 g for 10 min at room temperature. The supernatant was recovered and mixed with distilled water (1.0 mL) and 0.1% FeCl_3_ (150 µL). Then, the absorbance was measured at 700 nm. Higher absorbance of the reaction mixture (according to the blank) indicates greater reducing power. This determination was made according to a published protocol [69] with some modifications. The inhibition percentage (%I) was calculated according to the formula:

$$\%I=\frac{Ab-Aa}{Ab}\times 100$$

where *Ab* is the absorbance of the blank sample and *Aa* is the absorbance of the test sample.

The inhibition percentage was plotted versus the sample concentration to obtain the IC_50_ index.

#### 3.7.3. β-Carotene Blanching Test

The β-carotene/linoleic acid test evaluates the inhibitory effect of a compound or a mixture on β-carotene oxidation in the presence of molecular oxygen (O_2_) and gives an estimation of the antioxidant potential of the sample.

As previously described [70], a mixture of β-carotene and linoleic acid was prepared by adding together 0.5 mg of β-carotene, 25 µL of linoleic acid and 200 mg of Tween-40 in 1 mL of chloroform. The chloroform was then completely evaporated under vacuum and 100 mL of oxygenated water was subsequently added to the residue and mixed to form a clear yellowish emulsion. Then, 350 µL of various sample concentrations (200, 400, 600, 800, 1000 µg/mL) in methanol (extracts and ascorbic acid) was added to 2.5 mL of the above emulsion and mixed. The test tubes were incubated in a water bath at 50 °C for 2 h together with a negative control (blank) containing pure methanol instead of sample. The absorbance values were measured at 470 nm.

The antioxidant activity (percentage inhibition, % I) of the samples was calculated as follows:

$$\%I=\frac{A\;\left(\beta -carotene\;after\;2\;h\;assay\right)}{A\;\left(initial\;\beta -carotene\right)}\times 100$$

where *A (β-carotene after* 2 *h assay)* is the absorbance value of β-carotene remaining in the samples, after the 2 h assay whereas *A (initial β-carotene)* is the absorbance value of β-carotene in the freshly prepared standard solution. The activity was calculated as 50% inhibition concentration (IC_50_). All experiments were repeated three times on independent samples and the data are expressed as mean ± standard deviation (SD).

### 3.8. Anti-Malarial Activity

The anti-malarial activity was determined as previously described [71]. The asexual erythrocyte stages of *P. falciparum*, chloroquine-sensitive strain 3D7 were maintained in continuous in vitro culture, according the procedure of Trager and Jensen. The host erythrocytes were A+ human red blood cells obtained from a patient from Schiphol in the Netherlands (BEI Reagent Search) [72]. Crude extract solutions were prepared in DMSO (Sigma-Aldrich, Darmstadt, Germany, D-4540) at 10 mg/mL (or 1 mg extract diluted in 100 µL DMSO). The extract solutions were diluted 10 times in ready-to-use culture medium to give a 1 mg/mL solution. In a 96-well plate, each test sample was applied in a series of eight two-fold dilutions and tested in triplicate. Parasitemia was 2% and hematocrit was 1%, as described by Murebwayire et al. [73]. Infected red blood cells were used as a positive growth control and unparasitized red blood cells were used as a negative (blank) control. Artemisinin 98% (Sigma-Aldrich, Machelen, Belgium) at an initial concentration of 100 ng/mL was used as a positive control in all experiments. The plate was incubated for 48 h at 37 °C in a hermetically sealed culture dish impregnated with a GENbox microaer gasbag (bioMerieux, 96125) to generate a microaerobic medium. It was then kept at –20°C for 24 h after the 48-h incubation and thawed at 37 °C for 45 min. Then, 20 µL of each homogenized well was transferred to a new 96-well plate and 100 µL of a solution consisting of 1 mL Triton X-100 (Sigma, X100), 10 mg saponin (Merck, A18820), 1 g lithium L-lactate (Sigma, L2250), and 200 mg APAD (Sigma, A5251)/100 mL TRIS pH 8 buffer (Sigma, Darmstadt, Germany, T6664) was added. The new 96-well plate was incubated for 15 min at 37 °C, then 20 µL of a solution mixture prepared from 1 mL of a NTB solution (nitro-blue tetrazolium chloride; Sigma, Darmstadt, Germany, N6639) (2 mg/mL) in distilled water and 1 mL of a PES solution (phenazine ethosulfate; Sigma, P4544) (0.1 mg/mL) in TRIS pH 8 buffer were added protected from light and incubated for 30 to 45 min at 37 °C. Parasite growth was estimated by the determination of lactate dehydrogenase (LDH) activity, using the colorimetric method described in 1993 by Makler et al. [74,75]. Absorbance was measured with a spectrophotometer (Stat Fax 2100, Fisher, Illkirch, France) at 630 nm. The intensity of coloration is proportional to the amount of enzyme present in the reaction medium and, thus, to the amount of parasites. The IC_50_ values were calculated from the graphs.

### 3.9. Statistical Analysis of Biological Data

Data are expressed as means ± S.D; for the statistical analysis, ANOVA followed by Tukey’s test was used GraphPad Prism. Statistically significant differences were considered for *p*-values < 0.05. Pearson’s method was used to determine correlations.

### 3.10. Extraction, Isolation, and Characterization of Compounds

To better understand the origin of the reported biological activities of the leaf hydro-methanolic extract of *L. rhodesiensis*, the four flavones present in highest concentration in the methanolic extract were isolated and characterized. To do so, 1.0 kg of dry *L. rhodesiensis* leaves was ground and mixed with 15 L MeOH/H_2_O (50/50, *v*/*v*). The extract solution was concentrated under vacuum to give a dark-brown residue (122 g). Eighty-five grams of the residue was suspended in MeOH/H_2_O (850 mL). This solution was successively partitioned with solvents of increasing polarity such as hexane, dichloromethane (CHCl_3_) and ethyl acetate (EtOAc) (1.0 L: 2 × 500 mL each). The EtOAc fraction (22.7 g) was purified over a silica gel CC with stepwise CHCl_3_-EtOAc solvents (30:0 to 70:100) and stepwise EtOAc-MeOH solvents (95:0 to 5:100) to obtain eight subfractions (FAE.1-FAE.8), after combining the eluates on the basis of TLC (Thin-layer chromatography) analysis. Subfraction FAE.2 (2.5 g) was separated using preparative HPLC with an ACN-H_2_O + 0.1% HFO (20–40% ACN) solvent system for 25 min to afford compound **1** (4.8 mg). In addition, the CHCl_3_ fraction (10.7 g) was purified on a silica gel chromatographic column (CC) with a gradient of CHCl_3_-EtOAc solvents (70:0 to 30:100) and stepwise EtOAc-MeOH solvents (90:0 to 10:100) to obtain 24 eluates. Deposits were obtained from subfractions FD1p, FD2p, FD3p, FD5p, FD6p, FD7p, FD12p, and FD13p. Other subfractions (FDM.1-FDM.10) were obtained after eluates were combined according to the TLC analysis. The subfractions FD1p, FD2p and FD3p were separated using preparative HPLC with an ACN-H_2_O + 0.1% H_3_PO_4_ (20–100% ACN) solvent system for 25 min to afford compound **2** (16.7 mg) and compound **3** (7.0 mg). Subfraction FDM.5 was also subjected to preparative HPLC with an ACN-H_2_O + 0.1% H_3_PO_4_ (35–45% ACN) solvent system to yield compound **4** (13.0 mg)

The structures of the compounds were established by spectral analysis, mainly HR ESI-MS, Q-TOF, ^1^H, ^13^C and 2D-NMR (COSY, HSQC, and HMBC), as well as by comparing their spectroscopic data with those reported in the literature.

Compound **1** (yellow needles):

^1^H-NMR (MeOH-d4, 500 MHz) δ: 7.12 (2H, s, H-2′, H-6′), 6.81(1H, s, H-8), 6.59 (1H, s, H-3), 3.99, 3.95 (3H each, both s, OMe-7, OMe-4′). ^13^C-NMR (MeOH-d4, 126 MHz) δ: 182.8 (C-4), 165.1 (C-2), 154.4 (C-7), 150.6 (C-9), 150.9 (C-5), 148.5 (C-4′), 145.7 (C-5′), 138.5 (C-3′), 102.5 (C-3), 130.0 (C-6), 121.4 (C-1′), 107.3 (C-6′), 105.2 (C-10), 101.7 (C-2′), 90.4 (C-8), 55.5, 55.5 (OMe-7, OMe-4′). HR-ESI-MS *m*/*z* 346.9 ((M + H)^+^, 100%).

Compound **2** (white, amorphous powder):

^1^H-NMR (MeOH-d4, 500 MHz) δ: 7.12 (2H, s, H-2′, H-6′), 6.64*, 6.61* (1H, each, s, H-3, H-8), 4.00, 3.98, 3.94, 3.93 (3H, 6H, 3H, 3H, s, OMe). ^13^C-NMR (MeOH-d4, 126 MHz) δ: 180.8 (C-4), 164.17 (C-2), 159.09 (C-7), 153.69 (C-3′, C-5′), 153.48 (C-5), 152.73 (C-9), 141.53 (C-4′), 132.76 (C-6), 126.58 (C-1′), 106.19 (C-10), 105.38 (C-3), 103.96 (C-2′, C-6′), 91.00 (C-8), 61.15 (OMe-6), 60.98 (OMe-4′), 56,50 (OMe-3′, OMe-5′, OMe-7); HR-ESI-MS *m*/*z*: = 388.9 (M + H)^+^. An asterisk (*) means that the values may be interchanged.

Compound **3** (colorless needles):

^1^H-NMR (MeOH-d4, 500 MHz, d, ppm, J/Hz): 3.96 (3H, s, OMe-4′), 3.93 (3H, s, OMe-3′), 3.85 (3H, s, OMe-6), 4.00 (3H, s, OMe-7), 6.76 (1H, s, H-3), 6.89 (1H, s, H-8), 7.55 (1H, d, J = 2.2, H-5′), 7.14 (1H, d, J = 8.5, H-2′), 7.68 (1H, dd, J = 8.5; 2.1, H-6′). ^13^C-NMR (MeOH-d4, 126 MHz, d) 56.6 (OMe-4′), 56.8 (OMe-3′), 57.0 (OMe-7), 61.1 (OMe-6), 92.4 (C-8), 104.7 (C-3), 106.8 (C-10), 110.6 (C-5′), 112.8 (C-2′), 124.8 (C-1′), 121.7 (C-6′), 133.8 (C-6), 151.0 (C-4′), 154.1 (C-3′), 151.8 (C-5), 154.9 (C-9), 161.0 (C-7), 166.0 (C-2), 184.2 (C-4). Mass spectrum Q-TOF, C_19_H_18_O_7_*m*/*z* 358.1 (M + H)^+^, Wiley library score 97.65%.

Compound **4** (yellow needles):

^l^H-NMR (MeOH-d4, 500 MHz) δ: 7.52 (1H, d, H-2′), 7.54 (1H, dd, H-6′), 6.94 (1H, d, H-5′), 6.85 (1H, d, H-8), 6.67 (1H, d, H-3), 4.00, 3.97 (3H, s, OMe-7, OMe-4′). ^13^C-NMR (MeOH-d4, 126 MHz) δ: 182.9 (C-4), 165.0 (C-2), 154.4 (C7), 150.7 (C-3′), 150.6 (C-5, C-9), 148.1 (C-4′), 130.0 (C-6), 122.4 (C-l’), 120.4 (C-6′), 115.4 (C-5′), 109.2 (C-2′), 105.2 (C-10), 102.4 (C-3), 90.6 (C-8), 55.6 (OMe-7), 55.3 (OMe-4′). HR-ESI-MS *m*/*z* 331.0862 ((M + H)^+^, 100%), Q-TOF C_17_H_14_O_7_.

The antioxidant potential of all purified compounds was determined based on the DPPH method.

### 3.11. General Procedure for the Determination of Compounds

^1^H and ^13^CNMR (proton and carbon nuclear magnetic resonance) spectra were recorded in MeOH-d4 on a Bruker NEO 500 MHz spectrometer equipped with a cryoprobe. 2D experiments were performed using standard Bruker microprograms. The heteronuclear multiple bond correlation (HMBC) spectrum analysis allowed us to confirm the position of carbonyl, methoxy and C-H correlations. The heteronuclear single quantum coherence (HSQC) spectrum analysis allowed us to identify C-H correlations. LC/MS was carried out on a Thermo Scientific LTQ orbitrap XL mass spectrometer with an ESI source in positive mode with an RP select B LiChrospher 60 (250 mm × 4.6 mm, 5 μm) column. A chromatography Interchim puriFlash 4250 equipped with a Büchi fraction collector C-660 unit was used to accomplish the preparative isolation. An Agilent Technologies G1311B 1260 quant pump apparatus equipped with a PDA detector and a C18 column (Agilent, Santa Clara, CA, USA, Eclipse XDB-C18; 3.5 µm; 4.6 × 150 mm) were employed for analytical HPLC. HPLC-PDA determination of the chemical profiles of polyphenols in the studied extracts of leaves, stems, and roots were performed using a gradient of methanol and 0.05% trifluoroacetic acid (1 mL/min). Analytical TLC was used during the extraction and purification procedures in order to confirm the presence of polyphenol and flavonoid molecules in the different fractions. TLC was performed on pre-coated Silica gel 60 F254 (Merck, Hohenbrunn, Germany) plates. After development with EtOAc/formic acid/HOAc/H_2_O (100:11:11:26), the dried plates were sprayed with NP-PEG [natural product reagent (1% diphenylboryloxyethylamine in MeOH) and polyethylene glycol 4000 (5% polyethylene glycol 4000 in EtOH)]. The plates were dried again and examined under ultraviolet light (366 nm).

## 4. Conclusions

In the present study, different *L. rhodesiensis* organs were submitted to hydro-methanolic extraction and the antioxidant and anti-malarial activities of those extracts were evaluated. The present study showed variable results depending on the plant organ. Leaf and stem extracts showed an interesting phenolic compound content correlated with robust antioxidant and anti-malarial activities, while the root extract displayed lower activities. As antioxidant molecules are able to neutralize reactive particles called free radicals that are associated with inflammatory and painful phenomena, the antioxidant activities of *L. rhodesiensis* extracts support the claim regarding the traditional use of this plant for the treatment of various affections, such as rheumatism.

Hence, *L. rhodesiensis* is a potential source for isolating new exogenous antioxidant and anti-malarial molecules. Moreover, this is the first report on the in vitro anti-malarial activity of *L. rhodesiensis*. Four compounds were isolated from the *L. rhodesiensis* leaf extract. Compounds **1**, **3**, and **4** were reported for the first time in this plant. Compound **1**, which displayed the highest number of free hydroxyl groups on the benzene rings among all the purified molecules, had a high antioxidant potential, whereas compound **4** displayed an average potential. This study reported one new flavone isolated from the leaves of *L. rhodesiensis* (compound **1**). Further anti-malarial tests supported by bioassay-guided isolation of the active compounds in the leaf extract are suggested. Moreover, as the biological activities were highlighted here using in vitro assays, it is necessary to confirm them in vivo. In the next part of our study, we plan to evaluate the antioxidant and antimalarial activity of the isolated flavonoid compounds.

*L. rhodesiensis* is a plant that is widely present in tropical and sub-tropical regions. However, it should be noted that if the local population uses it extensively for its biological properties, it would be important to cultivate it in order to avoid its loss. Moreover, it would be interesting to study the variability in extract compositions and in the biological activities of plants grown in different locations and seasons, as it is known that the culture conditions can widely impact the production of secondary metabolites by plants.

## Figures and Tables

**Table:** 

Compounds	R_1_	R_2_	R_3_	R_4_
**1**	H	OH	OH	OH
**2**	H	OCH_3_	OCH_3_	OCH_3_
**3**	H	OCH_3_	OCH_3_	H
**4**	H	OH	OH	H

**Table 1:** Phytochemical screening of *L. rhodesiensis* organs.

Phytochemical Classes		Test Performed	Leaves	Stems	Roots
Polyphenols		Iron chloride 2%	+++	++	+
Flavonoids		Cyanidin	+++	+	+
Terpenes/sterols		Lieberman and Bürchard	++	+	++
Tannins	catechin	Stiasny	++	+	+
	gallic	Stiasny	++	++	+
Saponins		Foam formation	+	+	++
Alkaloids		Dragendorff	+	+	+
Leuco-anthocyanins		Cyanidin	-	-	-
Anthocyanins		Cyanidin	-	-	-

Note: (-): not detectable, (+): low amounts, (++): high amounts, and (+++): very high amounts.

**Table 2:** Polyphenolic compound assay results. GAE: gallic acid equivalents, QE: quercetin equivalents (mean ± standard deviation of three independent tests).

Polyphenolic Compound Contents		
	Total Polyphenols (mg GAE/g Extract)	Total Flavonoids (mg QE/g Extract)
Leaves	273.27 ± 0.48	110.54 ± 0.46
Stems	206.06 ± 0.87	52.95 ± 0.64
Roots	153.37 ± 0.61	34.87 ± 0.34

**Table 3:** Results of the anti-malarial activity of the different extracts obtained by non-sequential extraction (50% inhibition concentration, IC_50_).

Extract (MeOH/H_2_O)	3D7, IC_50_ (µg/mL)
Leaves	12.5 ± 2.5
Stems	˃100
Roots	˃100
Artemisinin	0.004 ± 0.001

**Table 4:** ^1^H and ^13^C-NMR spectroscopic data for compounds **1–4** (500 MHz, MeOD).

	1		2		3		4	
	*δ*_H_ m (*J* in Hz)	*δ*_C_	*δ*_H_ m (*J* in Hz)	*δ*_C_	*δ*_H_ m (*J* in Hz)	*δ*_C_	*δ*_H_ m (*J* in Hz)	*δ*_C_
**2**		165.1		164.1		165.3		165.0
**3**	6.59, s	102.5	6.64, s	105.3	6.76, s	104.1	6.67, s	102.4
**4**		182.8		182.8		182.6		182.9
**5**		150.9		153.4		151.8		150.6
**6**		130.0		132.7		131.7		130.0
**7**		154.4		159.1		159.4		154.4
**8**	6.81, s	90.4	6.61, s	91.0	6.89, s	92.7	6.85, s	90.6
**9**		150.6		152.7		154.9		150.6
**10**		105.2		106.2		105.4		105.2
**1’**		121.4		126.5		122.0		122.4
**2’**	7.12, s	101.7	7.12, s	103.9	7.14, d (8.5)	111.1	7.52, d (2.13)	109.2
**3’**		138.5		153.7		152.5		150.7
**4’**		148.5		141.5		151.0		148.1
**5’**		145.7		153.7	7.55, d (2.2)	108.8	6.94, d (8.3)	115.4
**6’**	7.12, s	107.3	7.12, s	103.9	7.68, dd (8.5–2.2)	119.9	7.54, dd (8.3–2.13)	120.4
6-OCH_3_			4.00, s	61.1	3.85, s	61.1		
7-OCH_3_	3.99, s	55.5	3.93, s	56,5	4.00, s	57.0	4.02, s	55.6
3’-OCH_3_			3.98, s	56,5	3.93, s	56.8		
4’-OCH_3_	3.95, s	55.5	3.94, s	60.9	3.96, s	56.6	3.99, s	55.3
5’-OCH_3_			3.98	56,5				

## Data Availability

Data is contained within the article.

## Sample Availability

Samples of the compounds are available from the authors in very small amount.
